# Association between continuity of primary care and preventable hospitalization in adults with asthma: A cohort study

**DOI:** 10.1371/journal.pone.0325553

**Published:** 2025-06-06

**Authors:** Sangwan Kim, Eunjung Choo, Eun Jin Jang, Nam Kyung Je, Iyn-Hyang Lee

**Affiliations:** 1 Department of Preventive Medicine, Seoul National University College of Medicine, Seoul, Republic of Korea; 2 College of Pharmacy, Ajou University, Suwon, Republic of Korea; 3 Department of Data Science, Andong National University Andong, Republic of Korea; 4 College of Pharmacy, Pusan National University, Busan, Republic of Korea; 5 College of Pharmacy, Yeungnam University, Gyeongsan, Republic of Korea; 6 Department of Health Sciences, University of York, York, United Kingdom; University of Porto, Faculty of Medicine, PORTUGAL

## Abstract

**Objective:**

Hospitalization often indicates deteriorating health, longer treatment times, and higher healthcare costs. This study aimed to investigate associations between continuity of care (COC) and asthma-related hospitalizations using a rigorous methodology.

**Methods:**

This retrospective cohort study was conducted using national health insurance claims data. The study included adults with a diagnosis of asthma between 2015 and 2016 in a primary care setting. The exposure was measured using continuity of care indices (COCIs) during the first two years after inclusion. Cohorts were categorized into two groups based on COCI levels. The primary outcome was the incidence of asthma-related hospitalizations, and the secondary outcomes were emergency department (ED) utilization, systemic corticosteroid use, and asthma-related medical costs.

**Results:**

A total of 24,173 patients were eligible for analysis, 13,212 of whom were continuously cared for by primary doctors (the continuity group), and 10,961 non-continuously (the non-continuity group). During a 2 year-follow-up period, 230 patients (1.74%) were hospitalized in the continuity group and 404 (3.69%) in the non-continuity group. After adjusting for confounding covariates, patients in the non-continuity group were found to be at significantly higher risk of hospital admission (adjusted hazard ratio (aHR)=2.04 [95% confidence interval = 1.73 ~ 2.41]). In addition, the risk of ED visits, systemic corticosteroid use, and costs were higher for patients in the non-continuity group (aHR = 2.26 [1.32 ~ 3.87], adjusted OR=1.58 [1.35 ~ 1.82], and exp^β^ = 1.41 [1.37 ~ 1.45], respectively).

**Conclusions:**

In adult asthma patients at the early stages of illness, increased continuity of primary care was found to be associated with fewer hospitalizations, fewer ED visits, and lower healthcare expenditures.

## Introduction

Hospitalization often signals worsening health, longer treatments, and higher healthcare costs. These costs go beyond medical bills, including lost productivity when patients or caregivers can not work. It also strains hospital resources and delays care for others. Therefore, it is crucial that hospitalizations be reduced whenever possible [1]. Admissions for conditions like asthma, which is categorized as an ambulatory care sensitive condition (ACSC), are considered preventable when effectively managed at the primary care level [1,2].

Health care continuity has been defined in several respects [3–6] and of these, relational continuity (also called inter-personal continuity) concerns long-term relationships between patients and their healthcare providers [3]. Relational continuity emphasizes the importance of the individual rather than the illness, and thus, highlights the core values of primary care [7]. van Loenen et al. concluded that improving access and continuity of care (COC) can reduce preventable hospitalizations [8] and while Huntley et al. identified four features affecting unscheduled secondary care: COC, access, practice features, and quality of care [9]. Long-term patient-provider relationships enhance communication, build trust, and improve chronic condition management by encouraging information sharing and better understanding of patients [3,7,10].

Several studies have demonstrated that high relational COC can improve the clinical conditions of patients [11–15] reduce secondary care utilization (e.g., hospital admission for ACSCs) [11,16–19] and lower medical costs [11,20,21]. On the other hand, low relational continuity negatively affects patient experiences [22,23]. Although evidence is accumulating for a wide range of conditions, the data linking high relational continuity to benefits in patients with specific conditions is still considered insufficient. A recent systematic review highlighted concerns that the current level of evidence is only low to moderate for asthma [12]. Moreover, recent studies have highlighted limitations in earlier analyses, such as the potential for reverse causality due to measuring exposure and outcomes simultaneously, and the classification of patients as having strong continuity based on only 2–3 visits over the study period, which blurs the relationship between continuity and outcomes [24,25].

Given this context, the present study was undertaken to examine the association between relational COC between adult asthma patients and primary care doctors on clinical and economic outcomes. We hypothesized that strong relational care continuity reduces hospital admissions and associated costs.

## Materials and methods

### Study design

This retrospective cohort study was performed and reported in accordance with the Strengthening the Reporting of Observational Studies in Epidemiology (STROBE) guidelines [26].

### Data sources

Anonymized national insurance claims data from 2014 through 2020, provided by the Korean National Health Insurance Service (KNHIS), were analyzed. The KNHIS database contains details of all claims made by Korean residents covered by National Health Insurance (NHI) and Medical Aid (MedAid) [27]. KNHIS data include de-identified patient socio-demographic information, diagnoses, and details of all medical services provided and medications prescribed [28]. Data analysis was performed in April 2023 ~ August 2024.

### Study timeframe

**Fig 1:**
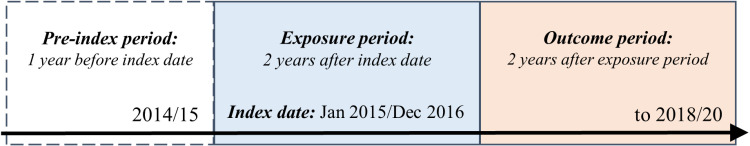
Schematic of the study periods.

The study period ranged from 2014 to 2020 (Fig 1). Index dates were defined as the dates of the first diagnosis of asthma during 2015–2016. The 12-month period preceding the index date was defined as the pre-index year. The exposure period was defined as the two years following the index date, and the outcome period was defined as the subsequent two years following the exposure period. Patients were followed from the end of the exposure period until outcomes were determined or data follow-up was lost, whichever occurred first.

### Definition of primary care

Continuity of primary care is conceptualized as the extent to which patient visits are concentrated among primary care doctors. However, the definition of primary care can vary across countries [29]. In this study, primary care is defined as community-based care, with inclusion criteria limited to clinics. Later, when measuring the Continuity of Care Index (COCI), visits to small and medium-sized facilities, excluding tertiary hospitals, were considered. In South Korea, patients benefit from universal coverage and are required to obtain a mandatory referral document only for tertiary hospitals. Consequently, if patients are located within the catchment area of a medium-sized hospital, they may receive ambulatory services at that facility. This situation contributes significantly to the weak gatekeeping function of conventional primary care in the Korean healthcare system [30].

### Study population

To minimize selection bias and maximize comparability, patients with asthma who were newly diagnosed in 2015−2016 at primary care clinics, aged 20 years or older on the index date, and who had made at least four ambulatory visits due to asthma during the exposure period were eligible for enrollment. Additionally, patients had to be prescribed at least one inhaler or patch for asthma on the index date. Asthma was identified based on the International Classification of Diseases, 10^th^ revision (ICD-10) using diagnosis codes J45-46. Individuals diagnosed with asthma in the year prior to the index date (the pre-index year) were excluded to ensure that only newly diagnosed patients were included. Additionally, those who visited tertiary facilities or experienced hospitalizations or emergency department (ED) visits during the exposure period were excluded. The final analytic cohort contained 24,173 unique patients.

### Measuring continuity of primary care

The Bice & Boxerman Continuity of Care Index (COCI) was used to measure COC [31]. Bice & Boxerman COCIs were calculated using the following formula [31,32].


$$COCI=\frac{\sum_{i=1}^{M}n_{i}^{2}-N}{N(N-1)}$$

Where N is the total number of visits made by a patient to any doctor, *n*_*i*_ is the number of patient visits to a specific provider *i*, and *M* is the number of potentially available providers.

The COCI is a dispersion index that is sensitive to the number of different doctors a patient visits. If a patient had 10 doctor visits, including nine visits with doctor A, their COCI would be 0.8. Alternatively, if a patient had eight visits with doctor A and two with doctor B, their COCI would be 0.64. Similarly, if a patient had eight visits with doctor A and one each with doctors B and C, their COCI would be 0.62. The COCI has a value of unity (1) if a patient always visits one specific health provider and a value of 0 if a patient never visits a single provider more than once.

### Outcome measures

The primary outcome measure was the incidence of asthma-related hospital admissions during the outcome period as ACSC hospitalization rates can serve as an indicator of primary care quality. Secondary outcomes were the incidence of asthma-related ED visits, systemic corticosteroid use, and asthma-related cost. Systemic corticosteroid use was defined as corticosteroid use in oral or injection form and used to evaluate asthma exacerbation. Asthma-related medical costs were defined as average annual medical costs, that is, the sum of the costs of claims identified using ICD-10 diagnosis codes J45 or J46 during the 4-year study period.

### Covariates

Covariates included individual characteristics such as sex and age. Patient NHI contributions were classified as high, moderate, or low and used as proxies of economic circumstances. The KNHI has two types of health programs: NHI and MedAid, and about 97% of the population is covered by NHI and 3% by MedAid [27]. Locations of residences on index dates were classified as large urban (metropolitan cities), small urban (other cities), or rural based on population densities. Elixhauser comorbidity indices (ECIs) were utilized as proxies of health statuses [33] and were based on diagnoses obtained from outpatient and inpatient records during the pre-index year. In addition, we considered whether patients had a diagnosis of allergic rhinitis, atopic dermatitis, hypertension, diabetes, dyslipidemia, cancer, osteoarthritis or rheumatoid arthritis during the pre-index year, or were being prescribed systemic corticosteroids during the exposure period. Frequent doctor visits were defined as outpatient clinic visits ranking at the 90^th^ percentile or higher during the exposure period. Disability status was designated ‘yes’ or ‘no’ on index dates.

### Configuration of the continuity cohort

Interim analysis revealed that patients with a COCI of 1 accounted for 54.7% of the study population (S1 Fig). As there is no generally used cut-off value for a high or low COCI, we assigned patients with a continuity score of 1 during the exposure period to the continuity group and all others to the non-continuity group. The mean study period was 3.94 (SD 0.29) years in both groups.

### Statistical analysis

A descriptive analysis was conducted on baseline characteristics and healthcare utilization. Inter-group comparisons of continuous and categorical variables were performed using the Kruskal-Wallis or Chi-squared tests, respectively. The adjusted hazard ratios (aHRs) and 95% confidence intervals (CIs) for the relationship between COC and hospital admissions or ED visits were assessed using a Cox proportional hazard regression model adjusted for baseline characteristics. A Kaplan-Meier graph was used to compare the continuity and non-continuity groups. Restricted cubic spline (RCS) plots were used to access relationships between continuous COCI and hospital admissions or ED visits [34,35]. Optimal cubic functions for the adjusted HRs of each 3^rd^ COCI percentile were estimated using COCI = 1 as the reference [36]. Systemic corticosteroid use was assessed using a logistic regression model, and results are presented as odds ratios (ORs) and 95% CIs. A gamma distribution and log link function were used to estimate asthma-associated costs, reported as exponential coefficients, while adjusting for covariates and average asthma-associated costs during the exposure period. Missing values were treated as an independent category in the statistical models. For testing the sensitivity of results, COCIs were calculated annually during the outcome period, using data from the previous two years, and analyzed with the generalized estimating equations (GEE) method to reflect the changing characteristics of COCI. Basic characteristics were subjected to subgroup analyses. Statistical analysis was conducted using SAS Ver. 9.4 (SAS Institute Inc, Cary, NC, USA), and, statistical significance was accepted for *p* values <0.05.

### Ethics statement

This study was performed in accordance with the Declaration of Helsinki and reviewed beforehand by the Institutional Review Board of Yeungnam University (IRB no. 7002016-E-2022-002-01), which waived the requirement for participant consent because the study was conducted using anonymous claims data provided for research purposes by the KNHIS.

## Results

### Baseline characteristics of the study population

**Fig 2:**
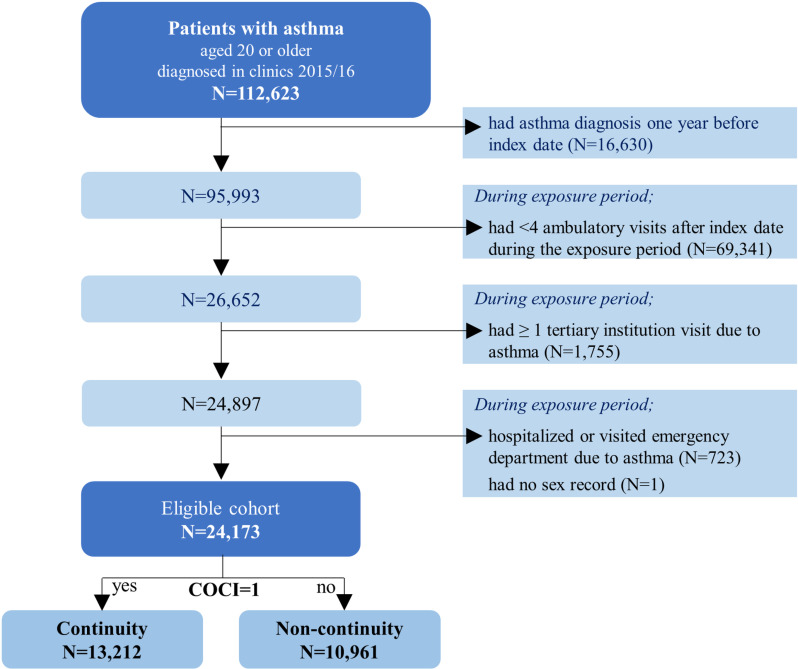
Selection of the study participants. COCI = continuity of care index.

The selection process is shown in Fig 2 and the baseline characteristics of the study patients are provided in Table 1. A total of 24,173 patients were included in the analysis.

**Table 1:** Baseline characteristics of study population.

Variable		All patients (N=24,173)	Continuity (N=13,212)	Non-continuity (N=10,961)	*p-value*
Sex	Women	16,229 (67.1)	8,887 (67.3)	7,342 (67.0)	0.6425
Age	mean±SD	50.6±16.7	51.2±16.3	50.6±17.1	0.0002
	20~ younger than 45	10,005 (41.4)	5,268 (39.9)	4,737 (43.2)	<0.0001
	45~ younger than 65	8,554 (35.4)	4,927 (37.3)	3,627 (33.1)	
	65 or older	5,614 (23.2)	3,017 (22.8)	2,597 (23.7)	
Insurance program	NHI	23,267 (96.3)	12,731 (96.4)	10,536 (96.1)	0.3346
	MedAid	906 (3.8)	481 (3.6)	425 (3.9)	
Insurance contributions^a^	High	8,485 (35.1)	4,719 (35.7)	3,766 (34.4)	0.0052
	Moderate	8,855 (36.6)	4,751 (36.0)	4,104 (37.4)	
	Low	6,648 (27.5)	3,657 (27.7)	2,991 (27.3)	
Rurality	Large urban area	15,542 (64.3)	8,712 (65.9)	6,830 (62.3)	<0.0001
	Small urban area	6,282 (26.0)	3,228 (24.4)	3,054 (27.9)	
	Rural area	2,349 (9.7)	1,272 (9.6)	1,077 (9.8)	
Disability	Yes	1,189 (4.9)	581 (4.4)	608 (5.6)	<0.0001
Elixhauser comorbidity index	mean±SD	2.4±1.8	2.4±1.8	2.4±1.8	0.9281
	1	10,229 (42.3)	5,545 (42.0)	4,684 (42.7)	0.0694
	2	5,357 (22.2)	3,002 (22.7)	2,355 (21.5)	
	3+	8,587 (35.5)	4,665 (35.3)	3,922 (35.8)	
Comorbidity	Allergic rhinitis	20,146 (83.3)	10,929 (82.7)	9,217 (84.1)	0.0045
	Atopic dermatitis	1,123 (4.7)	604 (4.6)	519 (4.7)	0.5480
	Cancer	1,145 (4.7)	633 (4.8)	512 (4.7)	0.6620
	Diabetes mellitus	3,803 (15.7)	2,068 (15.7)	1,735 (15.8)	0.7077
	Dyslipidemia	7,625 (31.5)	4,142 (31.4)	3,483 (31.8)	0.4780
	Hypertension	6,147 (25.4)	3,358 (25.4)	2,789 (25.4)	0.9596
	Osteoarthritis	5,806 (24.0)	3,129 (23.7)	2,677 (24.4)	0.1800
	Rheumatoid arthritis	932 (3.9)	532 (4.0)	400 (3.7)	0.1293
Systemic corticosteroids use during the exposure period		13,805 (57.1)	6,938 (52.5)	6,867 (62.6)	<0.0001
Frequent visitors90^b^		2,536 (10.5)	1,010 (7.6)	1,526 (13.9)	<0.0001

MedAid=Medical Aid; NHI=National Health Insurance; SD=standard deviation.

*Note*: The *p-values* are comparing the continuity and non-continuity groups and are calculated using a chi-squared test for categorical variables and Kruskal-Wallis test for continuous variables.

^a^Information for insurance contributions was missing for 185 (0.8%) patients in all patients; 85 (0.6%) in the continuity group; 100 (0.9%) in the non-continuity group.

^b^‘Frequent visitors90’ are patients who were in the 90^th^ percentile or higher based on the number of visits during the exposure period.

Mean patient age was 50.6 years (SD 16.7). The majority were women (67.1%), 64.3% resided in a large urban area, 35.1 and 36.6% were members of households with high or moderate insurance contributions, respectively, and 96.3% were NHI beneficiaries, which concurs with the national average. ECI scores showed asthma was accompanied by one or two chronic diseases in the majority of patients. In addition to diseases in the ECI framework, allergic rhinitis was present in 83.3% of the study subjects, and during the exposure period, 57.1% were prescribed a systemic corticosteroid. A comparison of the two continuity groups showed that patients in the non-continuity group were more likely to be younger, disabled, prescribed systemic corticosteroids, and frequent visitors of healthcare providers. The mean COCI of the study subjects was 0.73 during the 4 year study period (S1 Table), and mean COCIs of the continuity and non-continuity groups were 0.95 and 0.47, respectively.

### Risks of hospital admission

**Fig 3:**
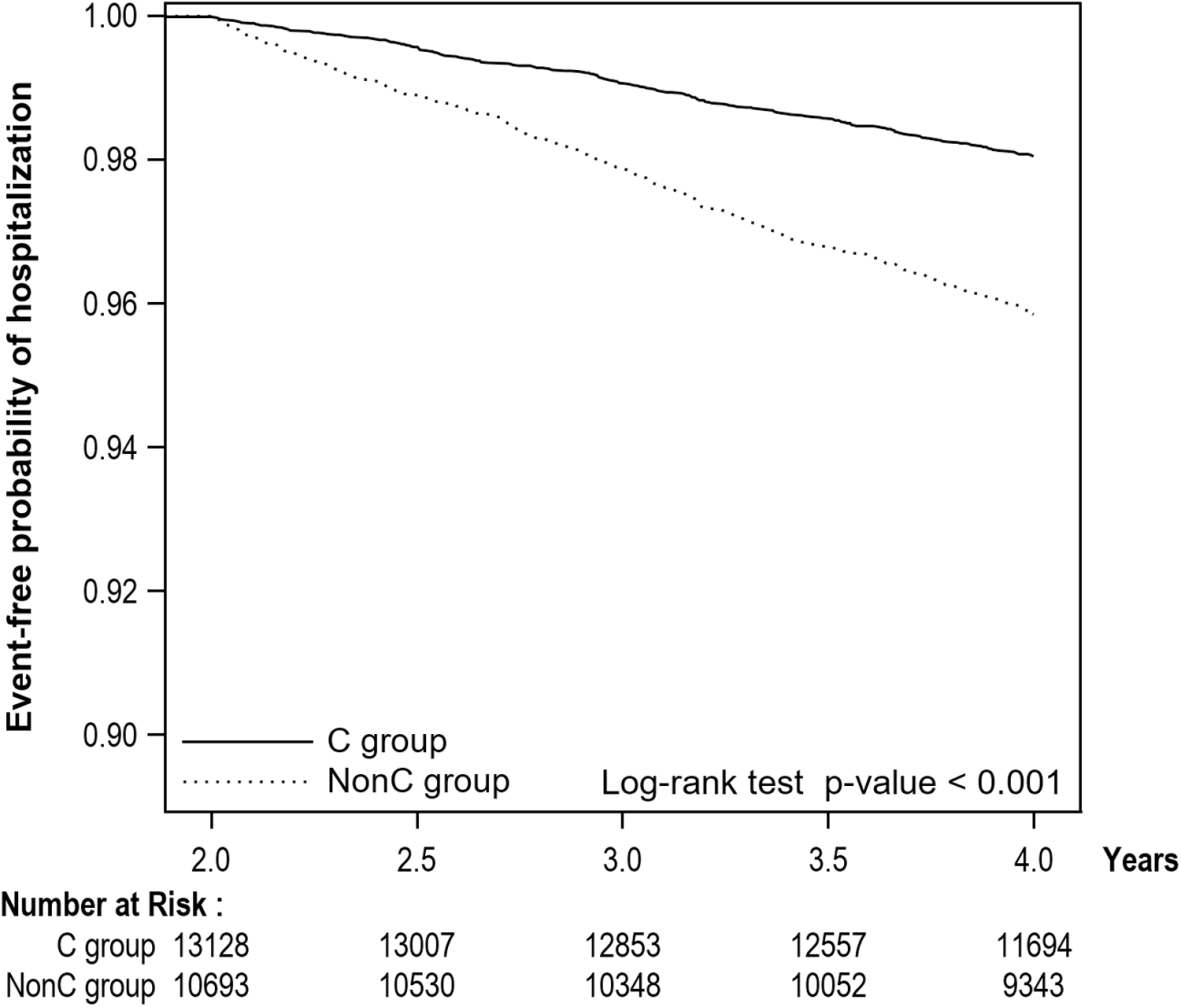
Kaplan-Meier curve of hospital admission during the outcome period by continuity of care. C = continuity; NonC = non-continuity.

**Fig 4:**
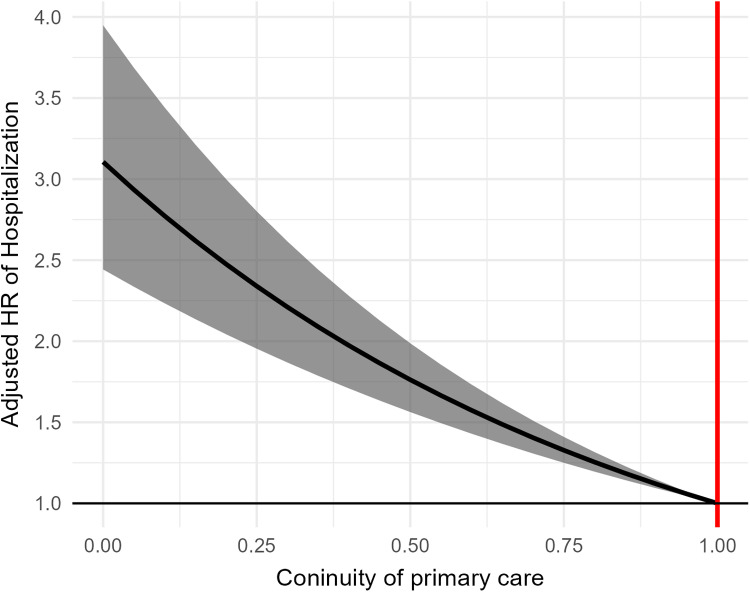
Relationship between adjusted HRs of hospital admission and continuity of primary care.

230 patients (1.74%) were hospitalized in the continuity group, and 404 patients (3.69%) in the non-continuity group for asthma-related reasons (Table 2). After adjusting for confounders, the non-continuity group had a significantly higher hospitalization risk than the continuity group (adjusted HR [aHR] = 2.04 [95% confidence interval = 1.73 ~ 2.41). The probabilities of patients avoiding hospital admission during the outcome period are shown in Fig 3. Sensitivity analysis revealed a slightly reduced risk of hospital admission in the non-continuity group (adjusted OR=1.46 [1.23 ~ 1.73]). Fig 4 shows the inverse relationships between COCIs and the aHRs of hospital admission.

**Table 2:** Descriptive summary of study outcomes and association of continuity of primary care with study outcomes.

Outcome	Continuity N = 13,212	Non-continuity N = 10,961	Measure of association	Unadjusted model (95% CI)	Adjusted model^a^ (95% CI)
				non-continuity (ref: continuity)	
Hospitalization, n (%)	230 (1.74)	404 (3.69)	HR	2.14 (1.82 ~ 2.52)^§^	2.04 (1.73 ~ 2.41)^§^
ED visit, n (%)	21 (0.16)	39 (0.36)	HR	2.26 (1.33 ~ 3.84)^†^	2.26 (1.32 ~ 3.87)^†^
Corticosteroid use, n (%)	415 (3.14)	419 (3.82)	OR	1.23 (1.07 ~ 1.41)^†^	1.58 (1.35 ~ 1.82)^§^
Asthma-related costs, mean±SD (1,000 KRW^b^)	139 ± 926	244 ± 1,315	exp^β^	1.76 (1.70 ~ 1.82)^§^	1.41 (1.37 ~ 1.45)^§^
**Sensitivity analysis using the generalized estimating equations method**					
Hospitalization			OR	1.53 (1.29 ~ 1.82)^§^	1.46 (1.23 ~ 1.73)^§^
ED visit			OR	1.55 (0.93 ~ 2.58)	Not available

CI = confidential interval; ED = Emergency department; exp = exponential; HR = hazard ratio; OR=odds ratio; ref = reference; SD = standard deviation.

^a^The adjusted HR and adjusted OR were analyzed after adjusting for covariates including sex, age, level of insurance contributions, health insurance program, rurality, disability, Elixhauser score, coexistence of allergic rhinitis, frequent doctor visitors and systemic corticosteroid use. ‘Frequent doctor visitors’ refers to individuals who ranked in the 90th percentile or higher based on the number of visits during the exposure period. In the analysis of medical costs, the annual medical cost during the exposure period was included as a covariate.

^b^1 US dollar = 1,500 KRW in January 2025

*p < 0.05;

† *p* < 0.01;

§ *p* < 0.001.

The demographic features that might be related to the increased risk of hospitalization were male sex, age of ≥ 45, living in a small urban or rural area, an ECI score of ≥ 3, and frequency of doctor visits (S2 Table). In particular, the aHR for hospitalization for those aged ≥ 65 was 5.92 [4.52–7.74] compared to the 20–44 age group.

### Secondary outcomes

#### Risk of ED visits.

Patients in the non-continuity group were more likely to visit an ED than those in the continuity group (aHR = 2.26 [1.32–3.87]).

#### Risk of systemic corticosteroid use.

415 patients (3.14%) in the continuity group and 419 patients (3.82%) in the non-continuity group were newly prescribed systemic corticosteroids during the outcome period (adjusted OR=1.58 [1.35–1.82]).

#### Asthma-related medical costs.

Mean annual asthma-related medical costs were significantly higher in the non-continuity group. After adjusting for major covariates, gamma regression modeling analysis showed that patients in the non-continuity group spent approximately 1.41 times more (1.37–1.45) than patients in the continuity group. The findings of secondary outcome measures are displayed in Table 2.

### Subgroup analyses on hospitalization and ED visits

The risk of hospitalization tended to increase in patients aged 20–44 in the non-continuity group, with significant variability observed. In patients with diseases of interest, e.g., allergic rhinitis, dyslipidemia, hypertension etc., the risk of hospitalization was not significantly different between those in the continuity and non-continuity groups (S2 Fig).

## Discussion

### Strengths of the study

The present study utilized a large, nationwide dataset representative of the South Korean population, which increases the trustworthiness of our findings. Furthermore, the data was analyzed using analytical techniques designed to address concerns raised in previous COC studies about the methods used. First, we separated exposure and outcome periods to eliminate the possibility of “reverse causality [13,24,25].” Second, to address concerns that condition severity might be a confounder [12], we included only new patients to minimize its potential impact. Third, to minimize the impacts of outliers, we set the inclusion criteria to at least four visits during the exposure period [25]. Additionally, we included patients whose number of visits was at the 90th percentile or higher as a covariate in the main analysis. The robust methodology used and the consistency observed between primary, sensitivity, and subgroup analyses strengthen confidence in our results.

### Principal findings

This study shows that a higher level of primary care continuity reduces preventable hospital admissions. More specifically, patients receiving non-continuous primary care had twice the risk of hospitalization, 2.3 times the risk of an emergency room visit, 1.6 times the use of systemic corticosteroids, and 1.4 times higher asthma treatment costs than patients receiving continuous primary care. Similarly, studies on asthma patients aged over 65 in Taiwan reported a 2.68 times higher hospitalization risk [37], and 2.1-fold increase in ER visits for patients that received non-continuous primary care [38]. In these Taiwanese studies, a low COCI was defined as < 0.47 ~ 0.5, which is similar to the definition used in the present; mean COCI in the non-continuity group was 0.47. A Korean study [39] that analyzed data between 2002 and 2006 showed that elderly asthma patients (65 ~ 84 years of age) in the non-continuity group had a similar risk of hospitalization (OR=2.07) and an ER visit (OR=2.25). In a study conducted in Quebec on asthmatic children aged 2 ~ 16, who maintained a continued relationship with their doctors, odds ratios (ORs) for hospitalizations and ED visits ranged from 0.66 to 0.82 and 0.8 to 0.92, respectively [40]. The findings of this study align well with the existing literature. In addition, Wireklint reported in 2021 that Swedish adults who maintained COC with their doctors were 2.19 times more likely to have sufficient knowledge to manage asthma exacerbations [41], which may partially explain why we found patients without COC had a higher rate of systemic corticosteroid use.

### Study limitations and future research opportunities

Several limitations should be considered. First, claims data analysis is observational, which introduces inherent limitations. For example, laboratory results, objective health measures, and patient behaviors (e.g., adherence medication, lifestyle factors) were not included. Second, the COC measurements were based on patient visit patterns and frequencies to primary care doctors, not directly measuring the quality of patient-doctor relationships [6,32,42]. Bias may arise from nonrandom doctor selection, complicating the distinction between COC effects and having a skilled doctor. However, continuity often reflects trust in a doctor’s skills (including communication skills), suggesting these concepts may be intertwined. Additionally, while short-acting beta agonist (SABA) use can serve as a useful indicator of mild asthma exacerbations and asthma control [43], we did not include it as a secondary outcome due to challenges in accurately assessing patient adherence and actual consumption from claims data. This limitation may have led to an underestimation of mild exacerbations that did not result in systemic corticosteroid prescriptions or emergency department visits. Furthermore, differences in routine prescribing patterns of asthma controller medications, such as inhaled corticosteroids (ICS) or ICS combined with long-acting beta agonists (ICS/LABA), were not analyzed. Assessing these medications could have provided further insights into the quality of regular asthma care across continuity groups. Third, the COCI is useful for assessing the effectiveness of care coordination and patient-provider relationships. However, there is ongoing debate regarding the criteria for determining the appropriate COCI threshold. Many previous studies analyzing patient outcomes have established empirical thresholds at which better health results are observed [11–21,24,37–40] and the present study is one such example. In a study that tested two COCI thresholds—0.8, a commonly used rule-of-thumb value, and the median, based on an analysis of the target data—the conclusions remained unchanged [25]. To better capture the dynamic nature of COCI over time, rather than restricting the measurement to the exposure period, it is necessary to conduct studies that track changes throughout the entire study period. Comparing the results of cohort grouping based on this approach with those derived from empirical methods would provide valuable insights. Fourth, the study has limitations in applying its findings to patients with an older diagnosis or those with very few visits. To minimize selection bias, we included newly diagnosed patients and those with at least four visits, ensuring similarity in patient characteristics and maximizing the comparability between the continuity group (exposed group) and the non-continuity group (unexposed group).

Lastly, caution is needed in generalizing the results to different healthcare contexts, particularly in terms of the organization of primary care, funding, and socio-cultural aspects. For example, this study defined primary care facilities based on the Korean context, which may limit comparability with results from other settings. Korea’s primary care reimbursement system primarily follows a fee-for-service model, which may present challenges when comparing with countries that use per capita funding models (e.g., the UK or certain European countries). Nonetheless, our findings align with studies in other healthcare settings, likely enhancing external validity and offering insights for countries facing similar challenges in sustaining robust primary care.

### Implications for practitioners and policy-makers

Despite evidence that COC is associated with better quality healthcare and reduced costs, COC is in decline at the frontline due to the increasing complexity of medical technology and organizations [44,45]. A dual strategy required to enhance COC in primary care. Frontline healthcare providers continue to strive to nurture continuous relationships with their patients, as ongoing relationships enhance mutual understanding and build trust, ultimately leading to a therapeutic alliance that embodies patient-centered care [13]. The ways primary care practices can improve COC were well suggested in Gupta and Bodenheimer’s opinion [46]. Two of the four practical strategies suggested in the study deserve particular attention. First, consistently assessing continuity of care at both the practice and clinician levels and making these metrics visible in relation to set goals can help staff track progress and enhance performance. Second, adopting team-based care models, where a small, consistent group of providers manages patient care, ensures continuity even when the primary doctor is unavailable. In addition to these strategies, a new approach involves strengthening electronic health records (EHRs) to improve team-based care. EHRs can facilitate seamless communication among providers, enhancing collaboration. Furthermore, fostering patient engagement through shared decision-making, regular follow-ups, and open communication is crucial for strengthening the doctor-patient relationship. Society should advocate for a healthcare environment that promotes continuity of care. While it is expected that the patient and doctor will develop a better match through continuous encounters, if that proves difficult, changing doctors may also be necessary. Switching doctors is not necessarily a negative thing and can even be positive if it leads to a better patient-doctor match. It is also important not to overlook the need to strike a balance between the two.

## Supporting information

S1 Table Continuity of care and annual health service utilization by period. (DOCX)

S2 Table Features associated with asthma-related events. (DOCX)

S1 Fig Continuity of care indices distribution. (TIF)

S2 Fig Risks of hospital admission by subgroup. (PDF)
