# Supplementary material for: Association between continuity of primary care and preventable hospitalization in adults with asthma: A cohort study
Source: PLoS One. 2025 Jun 6;20(6):e0325553. doi: 10.1371/journal.pone.0325553 (PMC12143515; doi:10.1371/journal.pone.0325553)
Supplement: S1 Table — (DOCX) [file pone.0325553.s001.docx]

**S1 Table. Continuity of care and annual health service utilization by period**

| **Variable** | | **All patients**  (N=24,173) | **Continuity**  (N=13,212) | **Non-continuity**  (N=10,961) |
| --- | --- | --- | --- | --- |
| *exposure period* | | | | |
| COCI | mean±SD | 0.77±0.29 | 1.00±0.00 | 0.48±0.19 |
|  | median (IQR) | 1.00 (0.50, 1.00) | 1.00 (1.00, 1.00) | 0.50 (0.35, 0.60) |
| Ambulatory visits | mean±SD | 7.8±6.6 | 7.2±5.6 | 8.7±7.4 |
|  | median (IQR) | 6 (4, 9) | 6 (5, 10) | 5 (4, 8) |
| Asthma-related costs (1,000 KRW^a^) | Total | 94±713 | 72±672 | 120±759 |
|  | Out-of-pocket payment | 19±107 | 13±74 | 26±136 |
|  | Public expenditure | 74±625 | 58±613 | 94±637 |
| *exposure and outcome period* | | | | |
| COCI | mean±SD | 0.73±0.29 | 0.95±0.15 | 0.47±0.19 |
|  | median (IQR) | 0.80 (0.47, 1.00) | 1.00 (1.00, 1.00) | 0.47 (0.33, 0.60) |
| Ambulatory visits | mean±SD | 10.5±10.6 | 9.3±9.0 | 12.0±12.1 |
|  | median (IQR) | 7 (5, 12) | 8 (5, 14) | 6 (5, 10) |
| Asthma-related costs (1,000 KRW^a^) | Total | 186±1,121 | 139±926 | 244±1,315 |
|  | Out-of-pocket payment | 36±179 | 25±122 | 49±229 |
|  | Public expenditure | 150±966 | 112±823 | 194±1,113 |

COCI=continuity of care index; IQR=interquartile range; KRW=Korean Won; SD=standard deviation.

^a^1 US dollar = 1,500 KRW in January 2025
