# Supplementary material for: Association between continuity of primary care and preventable hospitalization in adults with asthma: A cohort study
Source: PLoS One. 2025 Jun 6;20(6):e0325553. doi: 10.1371/journal.pone.0325553 (PMC12143515; doi:10.1371/journal.pone.0325553)
Supplement: S2 Table — (DOCX) [file pone.0325553.s002.docx]

**S2 Table. Features associated with asthma-related events**

| **Variable** | **Hospital admission** | | **ED visit** | |
| --- | --- | --- | --- | --- |
|  | **Adjusted HR**^*^  (95% CI) | ***p value*** | **Adjusted HR**^*^  (95% CI) | ***p value*** |
| Non-continuity (ref: Continuity) | 2.04 (1.73~2.41) | <0.001 | 2.26 (1.32~3.87) | 0.003 |
| Women (ref: men) | 0.58 (0.49~0.68) | <0.001 | 0.56 (0.34~0.94) | 0.027 |
| 45 or younger than 65 (ref: 20~44) | 1.82 (1.38~2.40) | <0.001 | 0.81 (0.44~1.48) | 0.493 |
| 65 or older (ref: 20~44) | 5.92 (4.52~7.74) | <0.001 | 0.56 (0.25~1.25) | 0.156 |
| MedAid (ref: NHI) | 1.28 (0.93~1.77) | 0.134 | 0.46 (0.06~3.54) | 0.456 |
| Moderate contribution (ref: high) | 1.13 (0.93~1.37) | 0.208 | 1.12 (0.62~2.02) | 0.718 |
| Low contribution (ref: high) | 1.19 (0.97~1.47) | 0.097 | 1.02 (0.51~2.03) | 0.951 |
| Small urban area (ref: large urban) | 1.28 (1.07~1.53) | 0.007 | 1.51 (0.83~2.73) | 0.177 |
| Rural area (ref: large urban) | 1.26 (1.01~1.58) | 0.042 | 3.39 (1.76~6.51) | <0.001 |
| Disability yes (ref: no) | 1.23 (0.97~1.57) | 0.092 | 0.64 (0.15~2.69) | 0.542 |
| ECI score 2 (ref: ECI score 1) | 1.32 (1.02~1.71) | 0.038 | 0.95 (0.47~1.90) | 0.878 |
| ECI score 3+ (ref: ECI score 1) | 1.76 (1.40~2.21) | <0.001 | 1.23 (0.65~2.35) | 0.522 |
| Allergic rhinitis yes (ref: no) | 0.87 (0.72~1.06) | 0.162 | 0.57 (0.32~1.01) | 0.056 |
| Systemic corticosteroids use yes (ref: no) | 0.97 (0.82~1.13) | 0.660 | 0.98 (0.58~1.66) | 0.946 |
| Frequent visitors90 (ref: else) | 1.62 (1.34~1.95) | <0.001 | 1.00 (0.45~2.23) | 0.995 |

CI=confidential interval; ECI=Elixhauser comorbidity index; ED=Emergency department; HR=hazard ratio; MedAid=Medical Aid; NHI=National Health Insurance; ref=reference.

^*^The adjusted HR was analyzed after adjusting for covariates including sex, age, level of insurance contributions, health insurance program, rurality, Elixhauser score, coexistence of allergic rhinitis and antihyperlipidemic agent use. ‘Frequent visitors90’ are patients who were in the 90^th^ percentile or higher based on the number of visits during the exposure period.
