# Supplementary figures and images for: Association between continuity of primary care and preventable hospitalization in adults with asthma: A cohort study
Source: PLoS One. 2025 Jun 6;20(6):e0325553. doi: 10.1371/journal.pone.0325553 (PMC12143515; doi:10.1371/journal.pone.0325553)

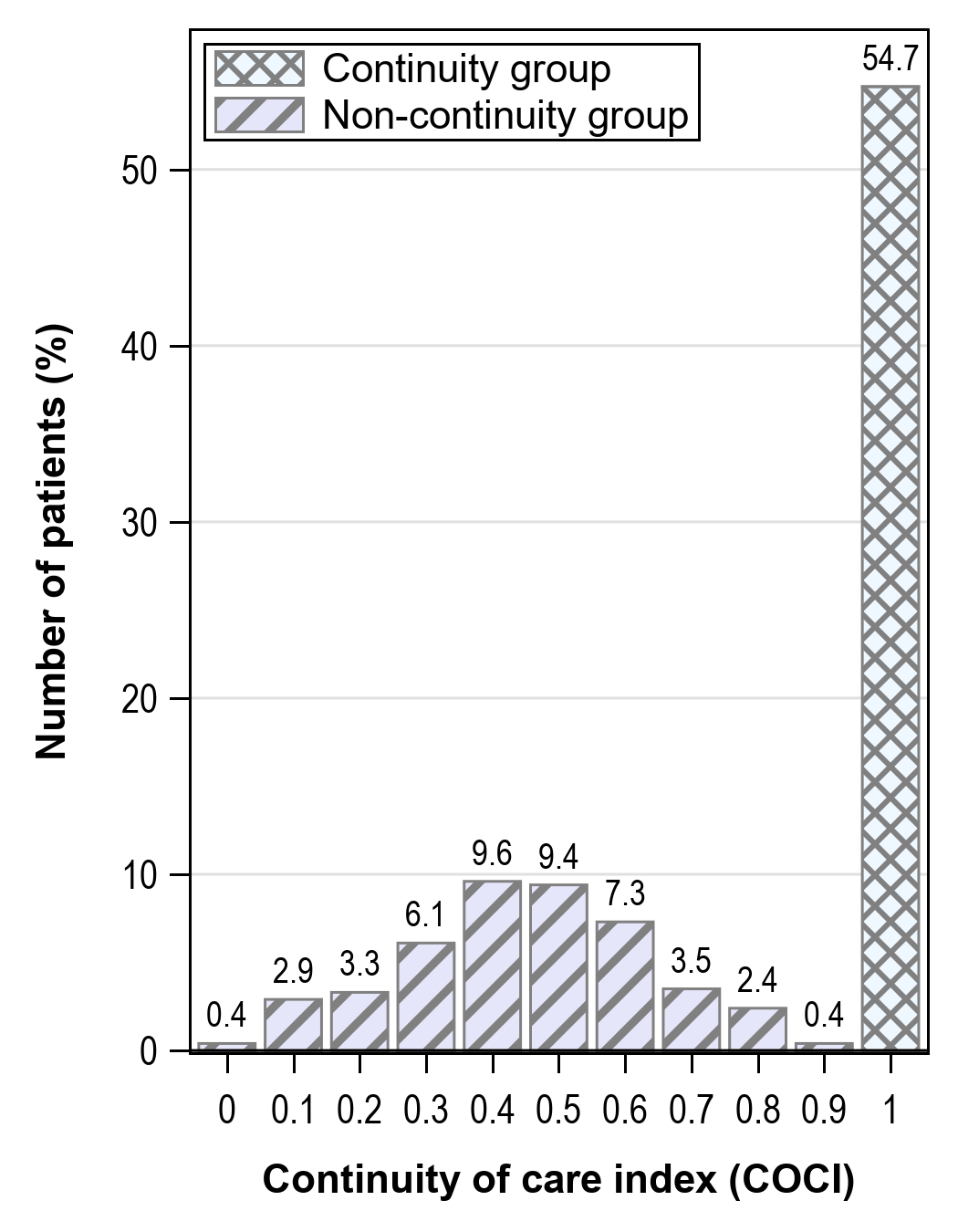

Supplement: S1 Fig — (TIF) [file pone.0325553.s003.tif]

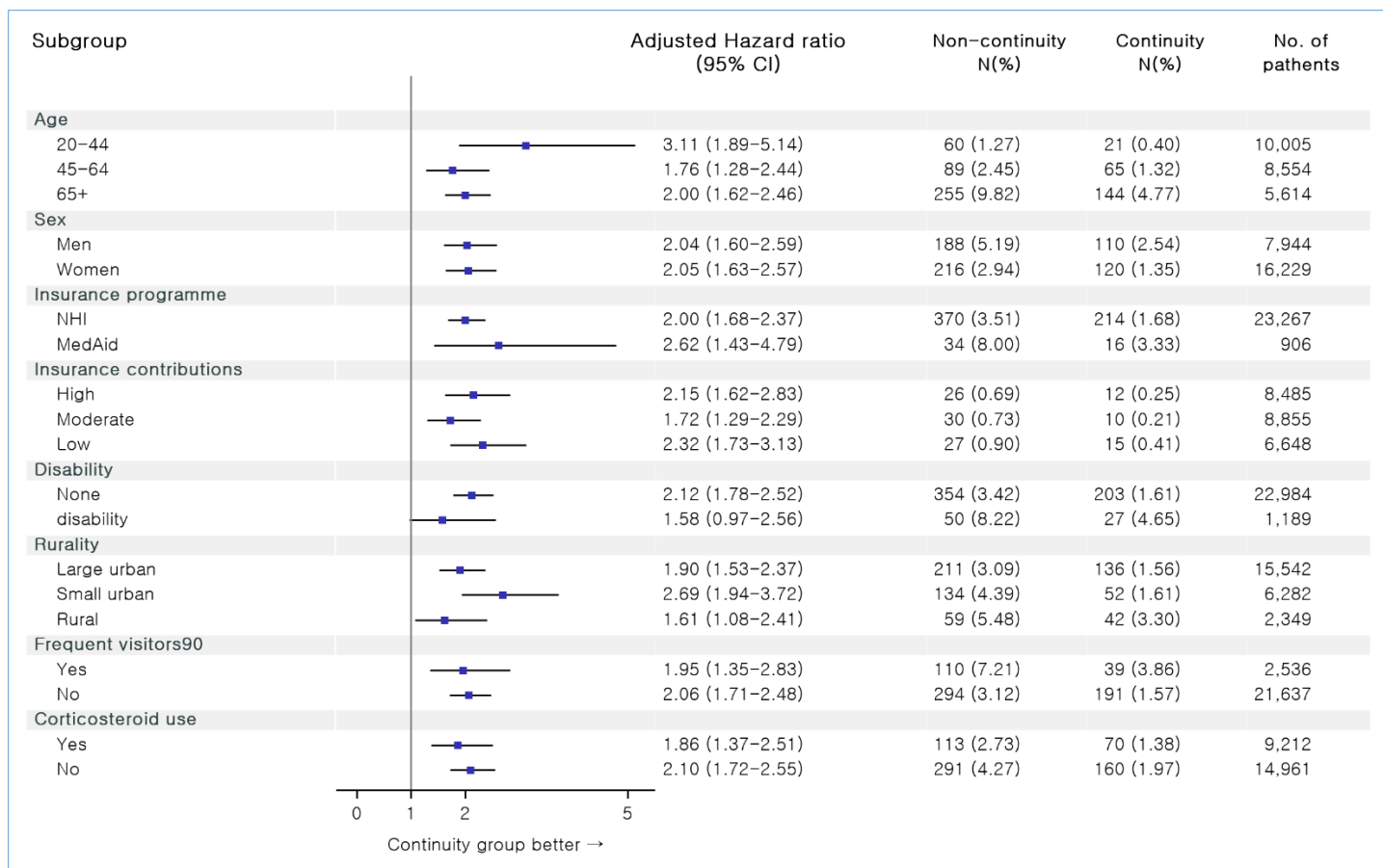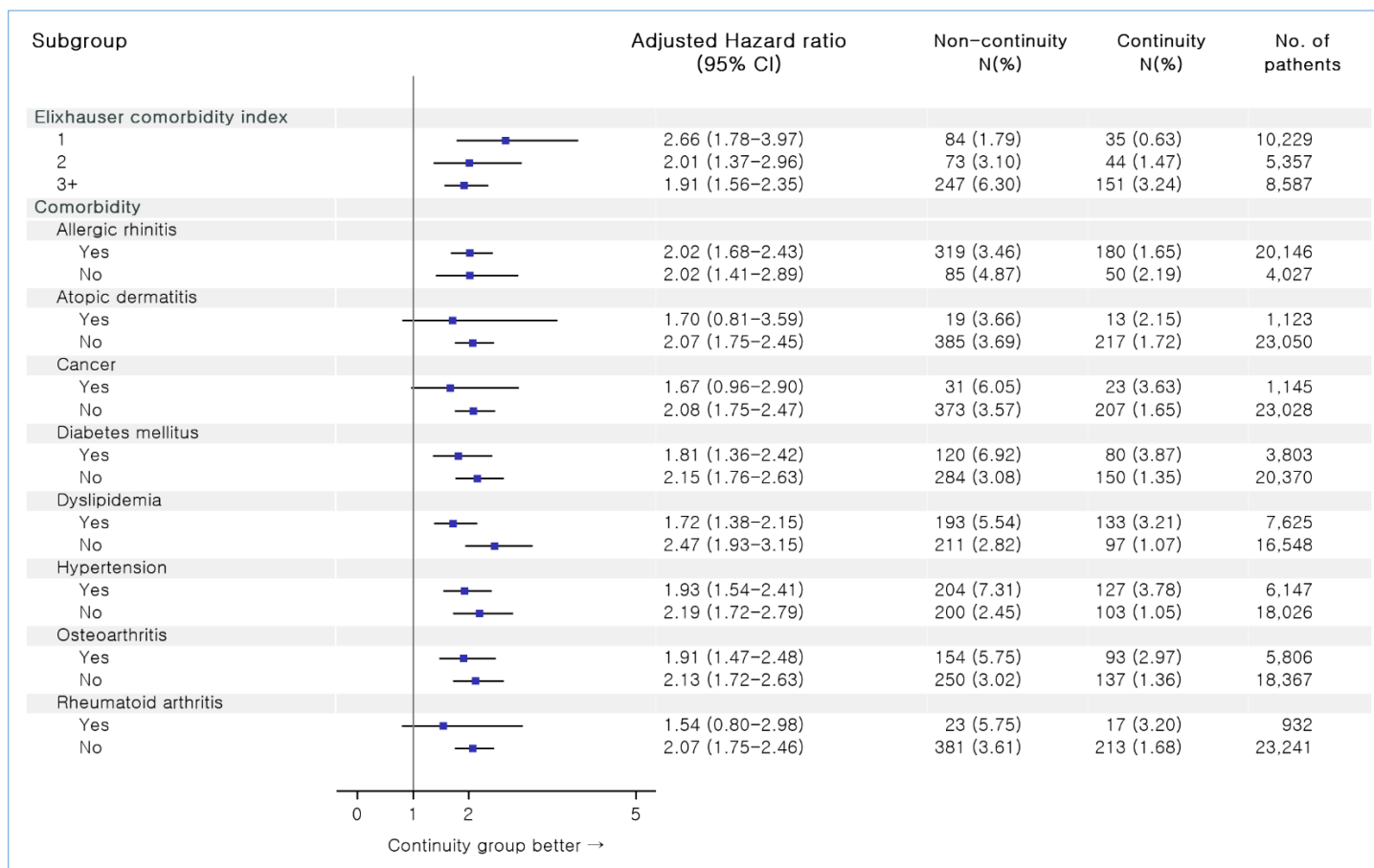

**S2 Fig. Risks of hospital admission by subgroup**

Supplement: S2 Fig — (PDF) [file pone.0325553.s004.pdf]
